# Measuring Snow Liquid Water Content with Low-Cost GPS Receivers

**DOI:** 10.3390/s141120975

**Published:** 2014-11-06

**Authors:** Franziska Koch, Monika Prasch, Lino Schmid, Jürg Schweizer, Wolfram Mauser

**Affiliations:** 1 Department of Geography, Ludwig-Maximilians-Universität München, Luisenstr. 37, Munich 80333, Germany; E-Mails: m.prasch@lmu.de (M.P.); w.mauser@lmu.de (W.M.); 2 WSL Institute for Snow and Avalanche Research SLF, Flüelastr. 11, 7260 Davos Dorf, Switzerland; E-Mails: lino.schmid@slf.ch (L.S.); schweizer@slf.ch (J.S.)

**Keywords:** GPS, low-cost, L-band, snow wetness, signal attenuation, permittivity

## Abstract

The amount of liquid water in snow characterizes the wetness of a snowpack. Its temporal evolution plays an important role for wet-snow avalanche prediction, as well as the onset of meltwater release and water availability estimations within a river basin. However, it is still a challenge and a not yet satisfyingly solved issue to measure the liquid water content (LWC) in snow with conventional *in situ* and remote sensing techniques. We propose a new approach based on the attenuation of microwave radiation in the L-band emitted by the satellites of the Global Positioning System (GPS). For this purpose, we performed a continuous low-cost GPS measurement experiment at the Weissfluhjoch test site in Switzerland, during the snow melt period in 2013. As a measure of signal strength, we analyzed the carrier-to-noise power density ratio (C/N_0_) and developed a procedure to normalize these data. The bulk volumetric LWC was determined based on assumptions for attenuation, reflection and refraction of radiation in wet snow. The onset of melt, as well as daily melt-freeze cycles were clearly detected. The temporal evolution of the LWC was closely related to the meteorological and snow-hydrological data. Due to its non-destructive setup, its cost-efficiency and global availability, this approach has the potential to be implemented in distributed sensor networks for avalanche prediction or basin-wide melt onset measurements.

## 1. Introduction

Seasonal snow is a very important reservoir component in the hydrological cycle, which releases temporarily delayed fresh water to the forelands. Downstream water suppliers are highly dependent on snow meltwater release from the alpine head-watersheds, which provides drinking and irrigation water, especially when mostly needed during the summer time. The average snow water equivalent (SWE) describes the total amount of snow stored within a certain catchment. However, this measure does not provide information on the state of snow melting. The liquid water content (LWC) *θ_w_* in snow describes the snow wetness of a snowpack. It is an indicator for snow melt and snow stability. A clear increase in liquid water leads to an onset of meltwater runoff within a catchment. This is relevant information, e.g., for flood predictions during intense melting due to intense solar radiation or rain-on-snow events. Temporal and quantitative meltwater delivery predictions are therefore highly demanded by decision makers in the field of water management dealing, e.g., with catchment runoff and flood forecasts [1–3], hydropower generation, as well as reservoir management [4,5] and, thereafter, optimization of hydropower production. Moreover, information on the LWC of a snowpack is important information for wet-snow avalanche forecasting, since infiltrating water affects the mechanical strength and hence the stability of the snowpack [6–8].

In general, snow wetness is difficult to measure *in situ*, as well as to derive from satellite-based remote sensing measurements. Moreover, to consider the spatiotemporal evolution of meltwater runoff and snow stability, there is a need for continuous, non-destructive monitoring of the LWC in snow. Changes in the LWC can quickly change snowpack properties and meltwater outflow [8,9]. They represent non-linear processes, which are difficult to detect or forecast. Manual snow wetness observations in snow pits only provide a rough estimate and are based on a wetness index [10]. An overview of several *in situ* snow wetness measurements is given by Boyne and Fisk [11] and Techel and Pielmeier [12]. Most *in situ* techniques are based on centrifugal, dielectric, dilution and calorimetric measurement methods. Instruments that measure the permittivity of wet snow are, e.g., the Denoth meter [13,14] and the Finnish Snow Fork [15]. They have in common that they are destructive, time consuming, laborious and can only be applied at accessible locations. Approaches of time-domain reflectometry (TDR) (e.g., [16]) were able to monitor the snow wetness continuously and non-invasively; however, this technique is also not applicable, e.g., in avalanche-prone areas. With upward-looking frequency modulated wave (upFMCW) and ground-penetrating radar (upGPR) systems, the bulk volumetric LWC can be derived also at avalanche starting zones (e.g., [17,18]). However, upward looking radar systems are limited in application to a single point and are expensive, power intensive and laborious to install.

The radiative properties of a snowpack in the range of L-band microwave radiation are mainly determined by the dielectric constant. Due to the high dielectric constant of water compared to dry snow, wet snow causes characteristic changes in microwave backscattering and penetration depth or emission rate regarding active or passive microwaves, respectively. Several microwave remote sensing approaches and their combinations were investigated for snow wetness detection (e.g., [19–24]). However, remote sensing applications are difficult to apply in alpine terrain, due to the low spatial resolution and complex topography (mountain shadowing and foreshortening effects). Because of the satellite's long repetition times, snow wetness parameters can only be detected every few days, depending on the swath width. Consequently, satellite remote sensing can currently not observe daily melt-freeze cycles. Moreover, regarding contemporary passive microwave systems, spatial resolutions up to 25 km^2^ are very coarse and not applicable for mountainous regions. In sum, microwave applications in alpine terrain lack the required temporal resolution to observe the spatiotemporal dynamics of important melt processes. Though various *in situ* measurements at the point scale and remote sensing approaches at a large scale have been investigated, it remains challenging to continuously and non-destructively determine LWC in snow with high spatial resolution.

Due to its global and continuous availability, new remote sensing applications based on the Global Navigation Satellite System (GNSS), such as the American GPS, the Russian GLONASS, the future European Galileo and the Chinese Beidou/COMPASS systems, have become increasingly popular. Several investigations were undertaken to detect snow depth or snow water equivalent based on multipath analysis of the carrier-to-noise power density ratio or carrier phase data [25–29]. An overview is provided by Botteron *et al.* [30] and Jin *et al.* [31]. These GNSS reflectometry techniques are mainly based on the analysis of reflections at the Earth surface in comparison to direct signals received at an antenna above the ground. Changes in snow depth [25,32], vegetation [33] or soil moisture [34] lead to changes in the reflected signals and signal polarization. Besides observations in the microwave range, surface reflectivity for snow surface monitoring is also applied at other wavelength spectral domains, like the near-infrared [35,36]. A detailed study on the reflectivity and polarization of GNSS signals was carried out by Najibi and Jin [37]. Besides snow depth estimates, Jin and Najibi [32] found that snow surface temperatures are detectable with dual-frequency GPS signals on behalf of reflected signals from the pseudorange and carrier phase. In general, these methods describe *in situ* measurements on a small scale covering an area of the GPS footprint according to the Fresnel ellipsoids, which depend on the antenna installation height above the reflecting surface and the elevation angle. According to Larson and Nievinski [38], the footprint can be up to hundreds of square meters. Schleppe and Lachapelle [39] showed with low-cost GPS receivers a clear signal attenuation within avalanche deposited snow and a decrease in position accuracy and precision due to pseudorange quality deterioration. Besides, ice loading on GPS antennas [40] and the effect of vegetation water content [41] were observed by signal attenuation approaches. However, to our knowledge, the existing GNSS snow studies were limited to flat terrain, and no studies regarding the snow wetness (LWC) have been carried out to date.

The aim of this study is to analyze whether the bulk volumetric LWC of an entire snowpack can be derived non-destructively at a high temporal resolution from GPS signal strength losses. We used low-cost GPS receivers and antennas, which are, in principle, globally applicable. The measurement devices are low power consuming; the data analysis is not time consuming and labor-intensive; and the measurements are taken in a continuous mode. After providing an overview of our new experimental low-cost GPS measurement setup at the Weissfluhjoch test site (2540 m a.s.l., Eastern Swiss Alps) (Section 2), we will describe the GPS data processing in Section 3. Signal changes due to atmospheric variability, reflection and refraction at the snow-atmosphere interface, as well as attenuation within the snowpack are considered in Section 4. In Section 5, the method to calculate the LWC based on signal attenuation is demonstrated and subsequently applied to the melt period in spring, 2013 (Section 6). The GPS data are shown in comparison with single reference measurements, as well as continuous meteorological and snow-hydrological data. The results are discussed in Section 7, and finally, Section 8 provides the conclusions and an outlook.

## 2. Measurement Setup

The flat test site, Weissfluhjoch (46°49′47″ N, 9°48′34″ E), is located above Davos (Eastern Swiss Alps) at an elevation of 2540 m a.s.l in a small, south-west facing valley. The site is well-equipped with numerous sensors to continuously record meteorological and snow properties [42]. It is an ideal location for GPS applications in high alpine areas, as it has a high satellite coverage, due to its almost unobstructed line of sight between the GPS receivers and the GPS satellites.

**Figure 1.**
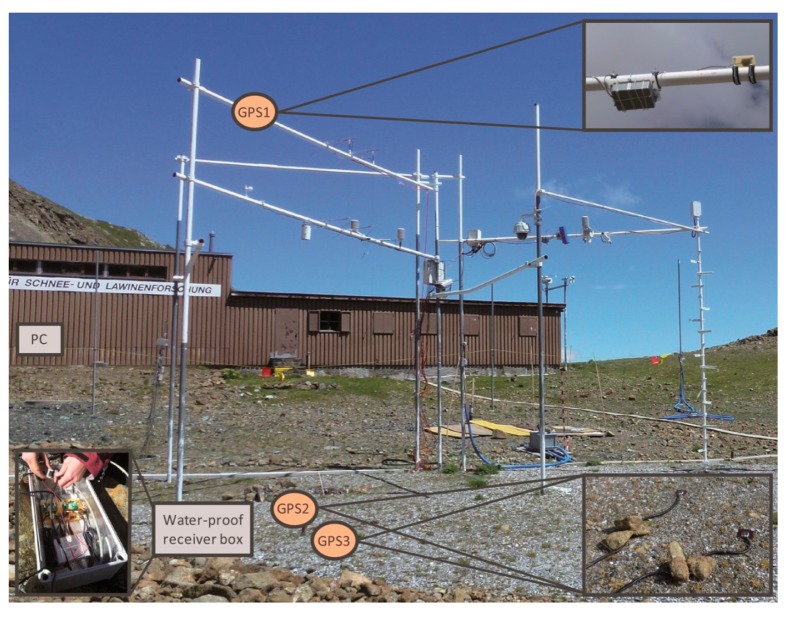
Overview of the location of the water-proof box with the Fastrax IT430 receivers, the hut where the PC was located and the Hirschmann GPS7M antennas of GPS1, GPS2 and GPS3 at the Weissfluhjoch test site on a snow-free reference day (26 September 2012).

On 26 September 2012, before the first snowfall in autumn occurred, we installed three low-cost Fastrax IT430 GPS receivers with SiRF IV chips [43], named GPS1, GPS2 and GPS3 (Figure 1). Each of the receivers was connected with low-cost Hirschmann GPS7M magnetic mount antennas [44]. The cost of each receiver and antenna pair (without data logging, data transmission, power supply, *etc*.) is approximately 150 USD. The antenna of GPS1 was mounted facing-up at a 4-m high pole approximately in the middle of the test site, to always be situated above the snow cover. Due to its small size of 3.9 cm × 3.9 cm and almost permanent wind influence at this exposed point, there was no or only negligible snow deposition on top of this antenna. Because GPS1 stayed snow-free, it delivered information on atmospheric influences on the GPS signals. The antennas GPS2 and GPS3 were installed also facing-up on the ground nearby GPS1. They stayed buried underneath the snowpack during the entire snow-covered period. The horizontal distance between GPS2 and GPS3 was approximately 1 m. The cable length between the receivers and the antennas was 3 m. Figure 1 illustrates the location of all three GPS antennas at the test site Weissfluhjoch on a snow-free day. The GPS antennas were connected to the receivers, which were stored in a weather-proof box, via 3-m coax cables. Between the weather-proof box and the data acquisition on a PC in the nearby hut, 70 m of power and data cable were installed. The GPS receivers recorded the raw data each second, which resulted in a total data volume of approximately 700 MB/day.

For validation, continuously measured meteorological and snow-hydrological data were analyzed, including air and snow surface temperature, snow depth measurements from a laser sensor and meltwater outflow at a snow lysimeter. The comprehensive set of meteorological and snow-hydrological measurements was aggregated to a resolution of half an hour. On eleven days, conventional manual snow profiles according to Fierz *et al.* [10] were recorded near the GPS system setup. In addition, the LWC was measured using the dielectric devices, Denoth meter and Snow Fork. The Denoth meter data were collected on six and the Snow Fork data on four dates during the considered spring period, on some days even at two different times. Both measurements were taken in the snow pit for each layer, and for analysis, the weighted mean for the entire snow depth was calculated. The snow profiles included an estimate of snow wetness for each layer (Wetness Index 1 to 5). To make this information comparable to the bulk volumetric LWC derived from the GPS recordings, we converted the wetness index of each layer to an approximate volumetric LWC (Wetness Index 1 to 5 corresponding to *θ_w_* of 0, 1.5, 5.5, 11.5 and >15%, respectively). The bulk volumetric LWC for the entire snowpack was calculated as the weighted mean of the LWC of each layer. The test site was continuously covered with snow from 27 October 2012, to 11 July 2013; the snow cover remained predominantly dry until mid-April. For the derivation of liquid water in snow, a three-months spring period (7 April–6 July 2013) was chosen as a representative demonstration period. The first week of this period is one week before the snow melt started and represents almost dry conditions, whereas during the rest of this period, the snowpack was mainly moist or wet.

## 3. GPS Raw Data and Processing of Normalized C/N_0_ Values

### 3.1. GPS Raw Data

The applied low-cost Fastrax IT430 GPS receivers track the L1 C/A code only. The freely available GPS broadcast is transmitted via microwaves at the L1-band at a frequency *f* of 1.57542 GHz [45]. The main field of application for low-cost GPS L1-band receivers is navigation, with its focus on positioning data. However, for deriving the LWC in snow, the received signal strength is of interest. As a measure, the signal strength is expressed as the carrier-to-noise power density ratio (C/N_0_). C/N_0_ is a bandwidth-independent index and is used for assessing signal quality. It quantifies the signal power of the received signal of tracked satellites [45].

Daily variations of the receiver noise due to temperature changes at the receiver and the cables were neglected, and the receiver noise was kept constant for the further modeling of the GPS input data. During the measurement period, GPS2 and GPS3 were always underneath the snow cover with a snow temperature at the bottom of the snowpack constantly at about 0 °C. At the upper antenna (GPS1), slight daily variations might occur, which were, however, neglected for the scope of this study. The ratio C/N_0_ is determined for each satellite that is in sight of the receiver at a certain time. At the Weissfluhjoch test site eleven GPS satellites can be tracked on average with the applied Fastrax IT430 receivers. In total, the system operates 32 GPS satellites grouped in six orbits with an equatorial inclination of 55 degrees. During a sidereal day (∼23 h 56 min), all GPS satellites can be tracked for several hours at the test site, Weissfluhjoch.

**Figure 2.**
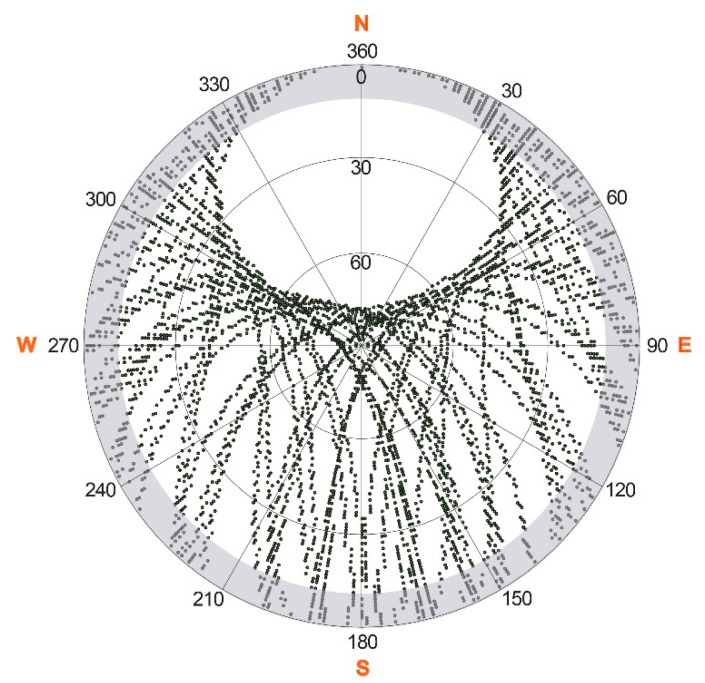
Sky plot as a polar plot of all GPS satellites recorded simultaneously by the three GPS receivers at the Weissfluhjoch test site for one sidereal day. The angular coordinates describe the azimuth angle and the polar axis the elevation angle. C/N_0_ values below an elevation angle of 10° were masked out for the calculation (these areas are marked grey).

For the study period (7 April–6 July 2013), the C/N_0_ data, logged in a receiver-dependent raw data protocol, were extracted for each of the 32 satellites at each time step of one second; in addition, the corresponding satellite number expressed as pseudo random noise (PRN), the time, as well as the satellite elevation and azimuth information were recorded. Due to a failure of GPS satellite PRN 27, there were no data available from this satellite during the observation period. The sky plot in Figure 2 displays the trajectory of all GPS satellites on their sky paths and illustrates the hemispherical coverage during one sidereal day at the test site, Weissfluhjoch. This pattern of trajectories is repeated every sidereal day and is identical for GPS1, GPS2 and GPS3. Due to the orbital arrangement of the satellite tracks, the northern azimuth angles have less coverage, because the test site is situated at northern latitudes. For further data processing, C/N_0_ values below an elevation angle of 10 degrees were masked out, because only weak signals were recorded below this elevation angle, or no signals were received, due to a slight hemispherical obstruction of the GPS satellites by surrounding slopes. The location of the flat hut (see Figure 1) in the north-eastern sector at an approximate distance of 40 m from the GPS receivers had negligible influence on the line of sight between GPS receivers and GPS satellites. All three receivers maintained lock on all GPS satellites (except PRN 27) every day, even when the maximum snow depth and LWC values were reached.

### 3.2. Normalized C/N_0_ Values

**Figure 3.**
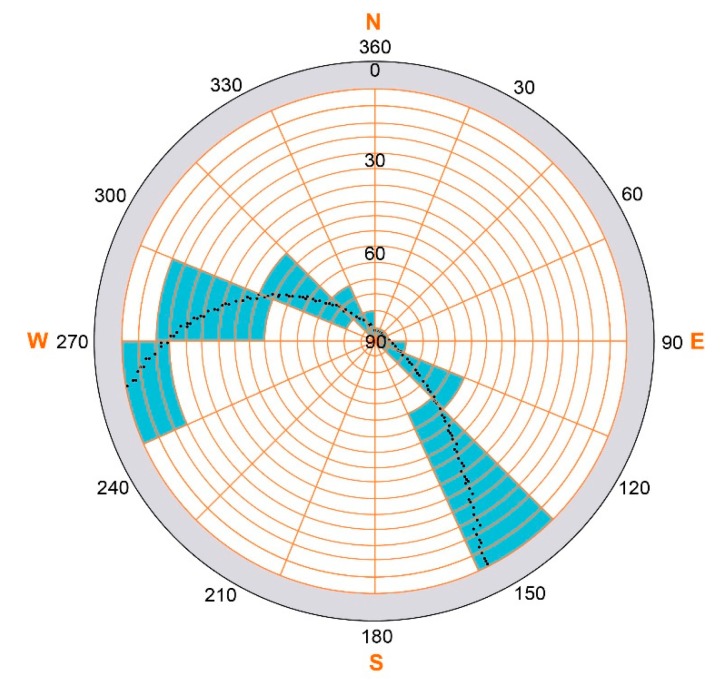
Sky plot as a polar plot for the GPS satellite PRN 1 for one sidereal day with the classification of the C/N_0_ values with 16 elevation and 16 azimuth classes. The classes assigned with values for PRN 1 are colored blue. C/N_0_ values below an elevation angle of 10° were masked out for the calculation (these areas are marked grey).

Besides environmental and atmospheric influences and the characteristics of receiver and antenna, the value of the recorded carrier-to-noise power density ratio (C/N_0_) depends on the elevation and azimuth angles of the received signals, as well as on the specific GPS satellite that emitted them. Due to different GPS satellite ages and other satellite characteristics, the peak field strength and, thereafter, also, the magnitude of the received C/N_0_ values differ slightly for each GPS satellite. Due to angle-dependent antenna sensitivity patterns, the received signal varies with different azimuth and elevation angles. Furthermore, multipath effects can cause changes in the received signals, especially at near grazing incidence. To account for all of these influences, several azimuth and elevation classes were defined to normalize the C/N_0_ values for each time step with respect to elevation- and azimuth-angle and satellite characteristics. In total, 16 elevation angle classes (in steps of five degrees between 10 and 90 degrees) and 16 azimuth angle classes for the entire azimuth range (in steps of 22.5 degrees) for all 32 GPS satellites were assigned, accounting for a total of 8192 classes. However, due to the hemispherical satellite distribution with less satellite tracks in the northern direction (see Figure 2), not all classes are covered by C/N_0_ values. Figure 3 shows exemplarily the C/N_0_ class distribution for GPS satellite PRN 1 for one sidereal day. One single class is covered only once by approximately 10 min. The mean class value is represented by the mean of all C/N_0_ values recorded during the satellite passage over this class.

To make the C/N_0_ values comparable individually for each GPS antenna, it was necessary to define a snow-free reference day encompassing the period of a sidereal day. Therefore, a representative sidereal reference day without a snow cover and stable and dry tropospheric conditions was chosen, which is, for this study, the UTC time period 09:00, 21 July 2013–08:56, 22 July 2013. It is assumed that for this time period, all C/N_0_ values were influenced by low atmospheric attenuation and ionospheric fluctuations. This means that all recorded C/N_0_ values during the entire time period theoretically represent the same signal strength. Thereafter, all values in each of the 8192 classes for this reference day express the normalized GPS C/N_0_ value 1.0. Then, all C/N_0_ values at each time step of each class were compared to the reference C/N_0_ value in the corresponding class. With this approach, also sub-daily variations of the C/N_0_ pattern, which are repeated every sidereal day, are considered and normalized. The data of all normalized C/N_0_ values calculated for each class with one-second resolution were aggregated to 30 min. Besides the temporal aggregation, the normalized GPS C/N_0_ values express also a spatial aggregation of GPS data recorded from different satellites at different elevation and azimuth angles covering different Fresnel zones around the antennas.

GPS1 as a snow-free reference measurement showed slight temporal fluctuations due to different atmospheric conditions for each time step, which impact also GPS2 and GPS3. During the snow-covered period, GPS2 and GPS3 were also influenced by the snow cover. The normalized half-hourly GPS C/N_0_ data of GPS1, GPS2 and GPS3 served as input for the further calculation of the LWC. Due to their almost identical location, GPS2 and GPS3 recorded the C/N_0_ values in parallel. For the observed three-month spring period, the coefficient of determination (R^2^) and the Nash-Sutcliffe efficiency coefficient (NSE) showed a very good agreement, with R^2^ = 0.96 and NSE = 1.00. For further data processing, for each time step, the average of the normalized GPS C/N_0_ from GPS2 and GPS3 was taken. The normalized C/N_0_ of GPS1 is denoted as *I*_m1_, and the normalized C/N_0_ of the mean of GPS2 and GPS3 is denoted as *I*_m2,3_.

## 4. Interaction of GPS Signals with the Snowpack

### 4.1. Overview of Processes and Influences on GPS Signals

**Figure 4.**
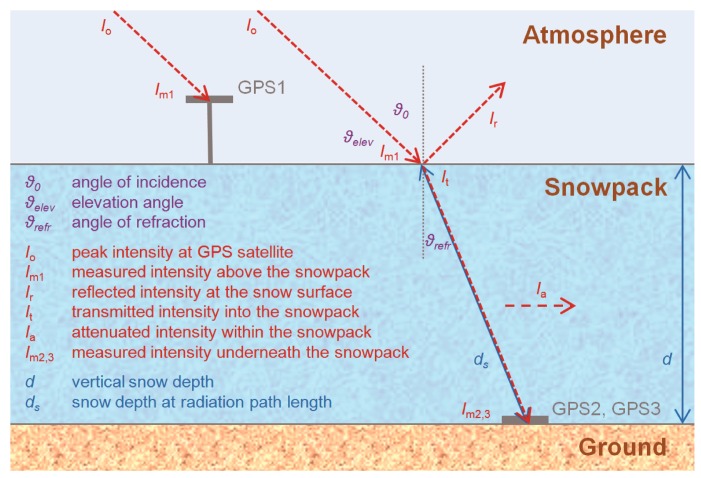
Overview of influences on the measured GPS intensity and radiation path length through snow, as well as the angle changes due to the reflection, refraction and attenuation processes. Only the direct signal paths are shown (in red); several multipath effects at and multiple bounces between the air/snow and snow/ground interfaces are neglected.

Several processes influence the GPS L1-band microwave signals on their way from the GPS satellites to the GPS receivers underneath the snow cover. These include reflection and refraction at the snow-air interface, as well as attenuation within the snowpack, which encompasses absorption and scattering [46,47]. These processes are mainly dependent on changes of the complex permittivity of a medium. For this study, ε*_s_* describes the complex relative permittivity of snow, which is given as:

(1) $${ɛ}_{s}={ɛ}_{s}^{\prime }+i{ɛ}_{s}^{\prime \prime }$$

where ${ɛ}_{s}^{\prime }$ is the real part and ${ɛ}_{s}^{\prime \prime }$ the imaginary part of the permittivity. An overview of received intensity and signal strength losses, as well as angle changes due to reflection, refraction and attenuation processes is given in Figure 4. The measured intensity *I_m_*_2,3_ underneath the snow cover at GPS2 and GPS3 can be described with:

(2) $$I_{m2,3}=I_{t}-I_{a}=I_{m1}-I_{r}-I_{a}$$

where *I_m_*_1_ is the received intensity at GPS1 above the snow cover, *I_r_* is the reflected intensity at the snow-air interface, *I_t_* is the transmitted intensity into the snowpack and *I_a_* is the intensity loss due to signal attenuation within the snowpack.

In general, signal losses in the atmosphere are much smaller than signal influences at the snow-atmosphere interface and within the snowpack. Comparing dry and wet snowpack conditions, a dry snowpack has less influence on the signals than wet snow. According to Mätzler [48], the penetration depth, defined as the distance at which the power is reduced to 1/*e* of its original value [47], of L-band microwaves in dry snow is up to 400 m, whereas at an LWC of approximately 1%, it decreases to ∼2.5 m and at approximately 5% to ∼0.5 m. The main reason is signal attenuation within the snowpack due to liquid water [19]. Due to the fact that water has a high complex permittivity compared to air and ice [46,49], the received signal power is remarkably lower for wet than for dry snow. The degree of refraction and reflection at the snow-atmosphere interface is also influenced by the LWC, but has a lower effect than losses due to attenuation within the snowpack. Due to a longer wavelength of GPS microwave radiation (L1-band: *λ* = 19 cm) compared to the approximately 10- to 20-times smaller size of snow grains, the effects of snow microstructure can be neglected in the range of L-band microwaves. In the following, the consideration of signal attenuation (including scattering and absorption), refraction and reflection in the processing chain are shortly described.

For the sake of simplicity, we neglected within our low-cost approach several multipath effects with coherent reflections at and bounces between the air/snow and snow/ground interfaces, which have only minor effects on the calculation of the LWC, as the latter is mainly based on the signal attenuation within the snowpack.

### 4.2. Atmospheric Influence and Variability

The incident radiation, also called peak intensity *I*_0_, which is sent directly from the GPS satellites, first undergoes losses by passing the atmosphere. These losses occur because of signal attenuation, reflection and refraction processes, e.g., at electrons, small particles and water vapor molecules in the ionosphere and troposphere, and can vary from day to day, due to different atmospheric and ionospheric conditions. These processes affect the incoming radiation at all three GPS receivers above and underneath the snow cover in the same way. For each time step, the intensity *I_m_*_1_ derived from GPS1 represents theoretically also the intensity that would be measured at GPS2 und GPS3 without the influence of the snowpack.

The normalized GPS C/N_0_ values received at GPS1 contain information about these atmospheric signal strength variations for each time step compared to the chosen reference day with good weather and stable ionospheric conditions. For all further calculations, the normalized GPS C/N_0_ values derived from GPS1 with its atmospheric variability information were used as input values to calculate the reflection, refraction and attenuation processes within the snowpack and at its surface.

### 4.3. Refraction and Snow Depth Correction

The calculation of the refraction influence on the GPS signals, expressed as the angle of refraction ϑ*_refr_* and the refraction coefficient *n*_s_, depends on the angle of incidence and the snow wetness. The mean angle of incidence at the GPS antennas at the Weissfluhjoch test site received from all GPS satellites considering all possible elevation angles between 10° and 90° is *ϑ*_0_ = 48° (which corresponds to a GPS elevation angle of *ϑ_elev_* = 42°). Due to:

(3) $$d_{s}=\frac{d}{\cos\vartheta_{\text{refr}}}$$

this oblique incident radiation travels a longer distance *d_s_* through the snow than the vertical distance that represents the snow depth *d* measured with the laser sensor. Refraction due to a change from one to another medium, such as at the snow-atmosphere interface, causes a change of the angle of incidence and, thereafter, also in *d_s_*. The refraction coefficient *n*_s_ of snow is described with:

(4) $$n_{s}=\sqrt{{ɛ}_{s}^{\prime }}$$

Because the refraction coefficient of the atmosphere can be approximated with 1.0, the angle of refraction *ϑ_refr_* at the snow-atmosphere interface is expressed using Snell's Law as:

(5) $$\sin\vartheta_{\text{refr}}=\frac{\sin\vartheta_{0}}{n_{s}}$$

The wetter the snow, the larger the value of ${ɛ}_{s}^{\prime }$ and, consequently, the higher the refraction. As a result, this means that *d_s_* decreases and gets closer to the measured snow depth *d*.

### 4.4. Reflection at the Snow Surface

Due to reflection at the snow-atmosphere interface, less than the entire intensity *I_m_*_1_ penetrates into the snowpack. As the incident radiation is circular polarized, the mean reflected intensity *I_r_* at one circumference (*α* = [0 2*π*]) is given by:

(6) $$I_{r}=\frac{\int_{0}^{2\pi }{\sqrt{{\left(r_{⊥}\sqrt{I_{m1}}\sin\alpha \right)}^{2}+{\left(r_{∥}\sqrt{I_{m1}}\cos\alpha \right)}^{2}}}^{2}d\alpha }{2\pi }=\frac{r_{⊥}^{2}+r_{∥}^{2}}{2}I_{m1}$$

with *r*_⊥_ and *r*_‖_ as the perpendicular and parallel reflection coefficients, respectively:

(7) $$r_{⊥}=\frac{E_{⊥}^{r}}{E_{⊥}^{i}}=\frac{Z_{s}\cos\vartheta_{0}-Z_{v}\cos\vartheta_{\text{refr}}}{Z_{s}\cos\vartheta_{0}+Z_{v}\cos\vartheta_{\text{refr}}}$$

(8) $$r_{∥}=\frac{E_{∥}^{r}}{E_{∥}^{i}}=\frac{Z_{v}\cos\vartheta_{0}-Z_{s}\cos\vartheta_{\text{refr}}}{Z_{v}\cos\vartheta_{0}+Z_{s}\cos\vartheta_{\text{refr}}}$$

with:

(9) $$Z_{s}=\sqrt{\frac{\mu_{0}}{{ɛ}_{0}\left({ɛ}_{s}^{\prime }+i{ɛ}_{s}^{\prime \prime }\right)}}$$

as the wave impedance for snow and:

(10) $$Z_{v}=\sqrt{\frac{\mu_{0}}{{ɛ}_{0}}}$$

as the free-space wave impedance (approximately corresponding to the wave impedance of the atmosphere), where *μ*_0_ is the magnetic constant and *ɛ*_0_ the electric field constant. $E_{⊥}^{i}$ and $E_{∥}^{i}$ are the field components of the incident wave perpendicular and parallel to the plane of incidence, respectively, and $E_{⊥}^{r}$ and $E_{∥}^{r}$ the field components of the reflected wave perpendicular and parallel to the plane of incidence, respectively.

### 4.5. Attenuation within the Snowpack

The attenuation coefficient *α* of a medium like snow is defined as:

(11) $$\alpha =\sqrt{\frac{\mu_{0}}{{ɛ}_{s}^{\prime }{ɛ}_{0}}}{ɛ}_{s}^{\prime \prime }{ɛ}_{0}2\pi f$$

and can additionally be described for a homogenous medium, as we assumed it for snow, by applying Beer-Lambert's law [50]:

(12) $$\alpha =-\frac{\ln\left(\frac{I_{t-}I_{a}}{I_{t}}\right)}{d_{s}}=-\frac{\ln\left(\frac{I_{m2,3}}{I_{m1}-I_{r}}\right)}{d_{s}}$$

with *I_m_*_1_ and *I_m_*_2,3_ as input data and *d_s_* and *I_r_* calculated with Equations (3) and (6), respectively. The attenuation within the snowpack increases with increasing snow wetness.

## 5. Calculation of Liquid Water Content Based on the Complex Permittivity

### 5.1. Real and Imaginary Part of the Complex Permittivity

Considering reflection, refraction and attenuation processes in the atmosphere, at the snow-atmosphere interface and within the snowpack, the bulk volumetric LWC based on the complex permittivity can be calculated by mixing formulas for wet snow. The complex permittivity of wet snow as a three-phase mixture of air, ice and water is dominated by its volumetric LWC. In general, wet snow has a significantly higher complex permittivity than dry snow [49,51]. Permittivity can either be estimated with an empirical relation [13,51] or three-phase mixing models (e.g., [52,53]). Most formulas for wet snow determination are valid within the pendular regime with an LWC below 8% to 10%.

Real part: Three approaches exist to determine the real part of the complex permittivity for wet snow [54] in the frequency range of the GPS signals: the empirical equations of Sihvola and Tiuri [15] and Denoth [13] and the three-phase mixing formula from Roth *et al.* [52]. With regard to the latter, Lundberg and Thunehed [54] found an empirical relationship for their measurements and showed similar results in calculating the LWC. The real part depends on both the snow wetness and the dry snow density [15,54]. The liquid water content (LWC) *θ_w_* is given as percent per volume.

(1) Empirical formula from Sihvola and Tiuri [15] after Tiuri *et al.* [51]:
  (13) $${ɛ}_{s}^{\prime }=1+1.7\times 10^{-3}\rho_{ds}+7.0\times 10^{-7}\rho_{ds}^{2}+8.7\times 10^{-2}\theta_{w}+7.0\times 10^{-3}\theta_{w}^{2}$$
  with *ρ_ds_* as the dry-snow density in kg/m^3^.
(2) Empirical formula from Denoth [13]:
  (14) $${ɛ}_{s}^{\prime }=1+1.92\times 10^{-3}\rho_{ws}+4.4\times 10^{-7}\rho_{ws}^{2}+1.87\times 10^{-1}\theta_{w}+4.5\times 10^{-3}\theta_{w}^{2}$$
  with *ρ_ws_* as the wet-snow density defined as:
  (15) $$\rho_{ws}=\rho_{ds}+0.01\theta_{w}\rho_{w}$$
  with *ρ_w_* = 1000 kg/m^3^ as the density of water.
(3) Three-phase mixing formula from Roth *et al.* [52]:
  (16) $${ɛ}_{s}^{\prime }={\left(0.01\theta_{w}{ɛ}_{w}^{\prime 0.5}+\frac{\rho_{ds}}{\rho_{i}}{ɛ}_{i}^{\prime 0.5}+(1‐\frac{\rho_{ds}}{\rho_{i}}‐0.01\theta_{w}){ɛ}_{a}^{\prime 0.5}\right)}^{2}$$
  with the permittivity of air ${ɛ}_{a}^{\prime }=1.0$, of ice ${ɛ}_{i}^{\prime }=3.18$ and of water ${ɛ}_{w}^{\prime }=88$ at 0 °C and a frequency of 1 GHz [8,54]. For this study, the dry‐snow density was assumed with *ρ_ds_* = 370 kg/m^3^, which was the bulk dry-snow density just before the first melting in the measurement period in spring, 2013, at the test site, Weissfluhjoch; the density of ice is *ρ_i_* = 917 kg/m^3^.

Imaginary part: The imaginary part ${ɛ}_{s}^{\prime \prime }$ of the complex permittivity for wet snow is directly related to the snow wetness and can be given according to Sihvola and Tiuri [15] after Tiuri *et al.* [51] and Bradford *et al*. [55] as:

(17) $${ɛ}_{s}^{\prime \prime }=\frac{f}{10^{9}\text{Hz}}(1.0\times 10^{-3}\theta_{w}+8.0\times 10^{-5}\theta_{w}^{2}){ɛ}_{w}^{\prime \prime }$$

with ${ɛ}_{w}^{\prime \prime }=9.8$ as the imaginary part of the complex permittivity of water at 0 °C and a frequency of 1 GHz [15].

**Figure 5.**
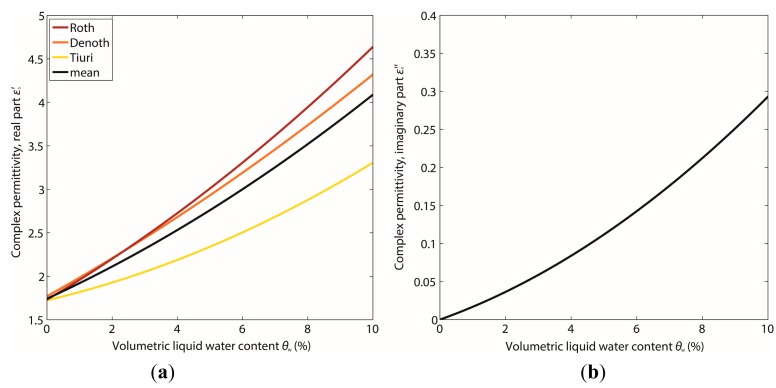
Permittivity *vs*. liquid water content (LWC) calculated with the three formulations and their mean for (**a**) the real part ${ɛ}_{s}^{\prime }$ and (**b**) the imaginary part ${ɛ}_{s}^{\prime \prime }$. The dry snow density was set to 370 kg/m^3^.

In the range of microwave radiation from 1 MHz–2 GHz [44], the real part of the complex permittivity of wet snow is frequency independent, so that these equations can be applied without corrections. However, the imaginary part depends on frequency in this microwave range. Figure 5 illustrates the dependence of the permittivity on the LWC for the different formulations (Equations (13), (14), (16) and (17)). The higher the LWC, the higher is the deviation between the different formulations.

### 5.2. Retrieval Algorithm

The input data for Equations (2)–(17) are the normalized GPS C/N_0_ data *I_m_*_1_ and *I_m_*_2,3_ and the snow depth *d* measured by a laser sensor nearby the GPS receivers every 30 min. With a root-finding algorithm, the two attenuation Equations (11) and (12) were set equal under consideration of the three different cases for the equations for the real part ${ɛ}_{s}^{\prime }$ of the complex permittivity of snow (Equations (13), (14) and (16)). The imaginary part ${ɛ}_{s}^{\prime \prime }$ was calculated after Equation (17). The permittivity was assumed to be temperature-independent, so no external temperature measurement was required as input. In summary, the only unknown of this approach is the LWC, which can be calculated as described above by the externally measured snow depth and the recorded GPS data for each time step assuming a constant value of the dry snow density (370 kg/m^3^), which means that it was held fixed at this nominal value. Table 1 illustrates how the reflectivity and the attenuation coefficient increase with increasing LWC; in particular, the attenuation coefficient strongly increases.

## 6. Results

The LWC of the snowpack covering the antennas GPS2 and GPS3 at the Weissfluhjoch test site was calculated for the three-months spring period (7 April–6 July 2013) in half-hourly temporal resolution. The system worked well apart from one break-down of 50 h from 19–21 June due to a power failure at the test site.

### 6.1. Bulk Volumetric Liquid Water Content Derived with the Three Different Formulations

**Figure 6.**
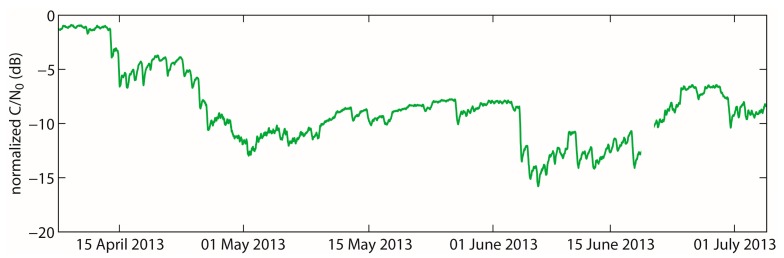
Normalized GPS C/N_0_ values (dB) taken as the mean of the two GPS receivers under the snow cover (GPS2 and GPS3) at the Weissfluhjoch test site for the period 7 April–6 July 2013.

Figure 6 shows the evolution of the normalized GPS C/N_0_ values in decibels taken as the mean of GPS2 and GPS3, which were buried under the snowpack during the entire spring period. These values serve besides the snow depth as main input data for the calculation of the LWC. Higher normalized GPS C/N_0_ (∼ −1 dB) values represent dry snow, whereas lower values (< −3 dB) represent moist to wet snow. A value of about 0 dB would represent snow-free conditions. The LWC was calculated as described above with the snow depth and the normalized GPS C/N_0_ values as input for each time step of 30 min. For the real part of the complex permittivity, the three approaches, in the following named Tiuri (Equation (13)), Denoth (Equation (14)) and Roth (Equation (16)), as well as their mean value were applied for each time step.

**Figure 7.**
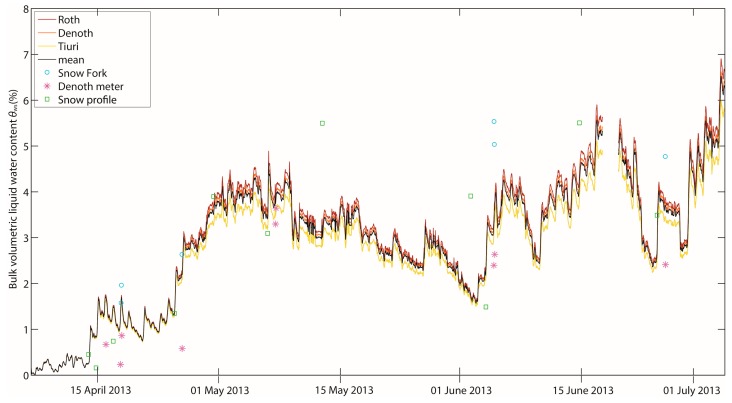
Bulk volumetric LWC of the snowpack above the antennas of GPS2 and GPS3 at the Weissfluhjoch test site for the period 7 April–6 July 2013, calculated with the normalized GPS C/N_0_ data. The LWC was calculated with three different formulations (Roth, Denoth and Tiuri). Furthermore, the mean of the three curves, as well as reference measurements with the Snow Fork, the Denoth meter and the estimate from manually observed snow profiles are shown.

The bulk volumetric LWC calculated as the mean of all three equations for the entire observation period covered a range from 0% to 6.3%. The minimum LWC determined with the three equations was 0%, and the maximum varied between 6% and 6.9%, with the lowest values calculated with Tiuri and the highest with Roth. In general, the differences in LWC between the different approaches increase with increasing LWC. The largest difference between the three mixing formulas was 0.9 percent points (pp). Figure 7 illustrates the temporal evolution of the bulk volumetric LWC calculated with the three mixing formulas for wet snow for the real part, as well as their mean value during the study period. In general, the evolution is fairly similar. At the beginning of the period, the snow was almost dry with a bulk volumetric LWC clearly below 0.5%. Around mid-April, the LWC started to increase and reached a first maximum of approximately 4% in the first week of May. Until the beginning of June, the values decreased to approximately 1.7% with a sharp increase in the first week of June up to a second peak, comparable to the level of the first maximum, *i.e.*, approximately 4%. The further increase until the beginning of July up to the maximum values was interrupted by three smaller minima around 2.5% to 3%. Moreover, all curves show small daily fluctuations of the bulk volumetric LWC indicating daily melt-freeze cycles, which we will focus on in more detail later. The differences due to the different formulations for calculating the LWC were quite small. In the following, to simplify comparisons, we will refer to the mean of the bulk volumetric LWC calculated from the three different formulations.

### 6.2. Comparison with Reference Measurements in Snow Pits

In addition to the GPS-derived LWC, Figure 7 includes single destructive reference measurements taken in snow pits nearby. These include measurements with a Denoth meter, a Snow Fork and wetness estimates for each stratigraphic layer according to Fierz *et al.* [10]. The wetness values determined with the Denoth meter and the Snow Fork did not match well with the GPS measurements. The under- and over-estimation compared to GPS-derived values was up to 2 pp. Moreover, values obtained with the Snow Fork were up to 3 pp higher than with the Denoth meter for the same date. However, this discrepancy is in accordance with previous findings by Techel and Pielmeier [12], Mitterer *et al.* [8] and Schmid *et al.* [18]. Overall, Figure 7 suggests that the values of the bulk volumetric LWC derived from the normalized GPS C/N_0_ data are approximately within the range of the values measured with the Denoth meter and the Snow Fork. During the first four weeks of the study period when the LWC was below 2%, the bulk values derived from the manually observed wetness index were slightly below the normalized GPS C/N_0_ curves. They agreed quite well at the end of the study period, but they clearly deviated in mid-May and at the beginning of June, when the LWC was generally high.

The root-mean-square deviation (RMSD) between the GPS-derived LWC and the reference measurements was about 1 pp (Table 2). The correlation between GPS-derived LWC and reference measurements was lowest for the snow profile estimates (adjusted R^2^ = 0.55).

### 6.3. Comparison with Meteorological and Snow-Hydrological Data

**Figure 8.**
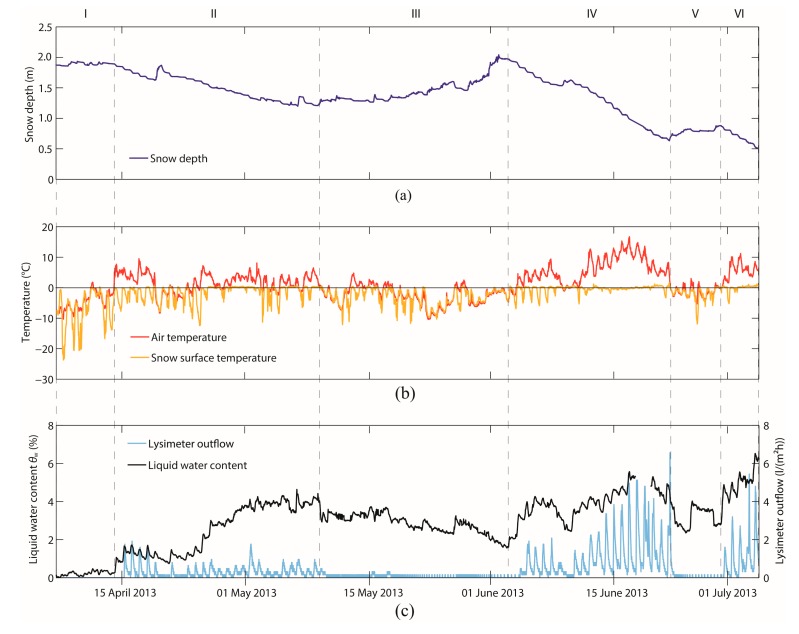
(**a**) Snow depth; (**b**) air and snow surface temperature and (**c**) lysimeter meltwater outflow, as well as the bulk volumetric LWC (mean of the three approaches, as in Figure 7) derived from normalized GPS C/N_0_ measurements at the Weissfluhjoch test site during the time period 7 April–6 July 2013. Vertical dashed lines between six sub-periods (denoted I to VI).

Figure 8 shows the evolution of the measured snow depth, air and snow surface temperatures and the meltwater outflow for a 5 m^2^ lysimeter, as well as the bulk volumetric LWC derived from the normalized GPS C/N_0_ measurements (only the mean is shown) at the Weissfluhjoch test site for the entire study period. In the following, the evolution is described by considering six sub-periods with different snow melt conditions.

Sub-period I (7–13 April 2013): This sub-period is characterized by almost dry-snow conditions. Due to cold air and snow surface temperatures, no discharge was registered at the lysimeter, and the bulk volumetric LWC was between 0 and 0.5%.
Sub-period II (14 April–8 May 2013): Due to melting, the snow depth decreased from 1.9 to 1.4 m. The air temperature, especially during mid-day, was mostly above 0 °C, and the snow surface temperature reached mostly 0 °C at its daily maximum, indicating snow melting processes. The LWC started to increase markedly on 14 April, one day before the start of the first lysimeter meltwater outflow, which indicates that the wetting front arrived at the bottom of the snowpack. The bulk volumetric LWC increased to approximately 4%, and the maximum daily discharge amounted to 1 to 2 L/(m^2^h).
Sub-period III (9 May–4 June 2013): Temperatures below 0 °C and several snowfalls causing the snow depth to increase to almost 2 m again characterized the third sub-period. These conditions resulted in an absence of meltwater outflow and a clear decrease in the bulk volumetric LWC. However, even though meltwater outflow stopped, the GPS-derived measurements suggested that approximately 2% of liquid water was still present in the snowpack at the end of this sub-period.
Sub-period IV (5–22 June 2013): The air temperature was almost always above 0 °C, reaching a maximum of up to 15 °C. Consequently, the snow surface temperature ranged, especially in the second week, around 0 °C. These melting conditions led to a strong decrease in snow depth and to an increase of the LWC of up to 5.5% and a peak discharge of up to 6.5 L/(m^2^h). The increase in discharge was temporally highly correlated with the increase in air temperature and LWC.
Sub-period V (23–29 June 2013): In contrast to the previous sub-period, temperatures were lower and no meltwater-outflow was registered. This sub-period was characterized by two small snowfalls, which resulted in a significant decrease in the bulk volumetric LWC, in particular during the snowfalls.
Sub-period VI (30 June–6 July 2013): Temperatures rose again, leading to a clear decrease in snow depth and an increase in meltwater outflow. The bulk volumetric LWC increased in parallel. After 6 July, the snow depth was spatially too variable to calculate reliable values of LWC. Furthermore, the snow wetness reached a higher bulk volumetric LWC than expected within the pendular regime, for which the applied mixing formulas are valid. The snow finally disappeared on 11 July, only five days after the end of this sub-period.

From the above descriptions, it becomes clear that the meteorological and snow-hydrological evolution had a great influence on the evolution of both the measured LWC and the lysimeter meltwater outflow; and that, in general, the temporal evolution of GPS-derived LWC agreed well qualitatively with meteorological, as well as the snow-hydrological data.

**Figure 9.**
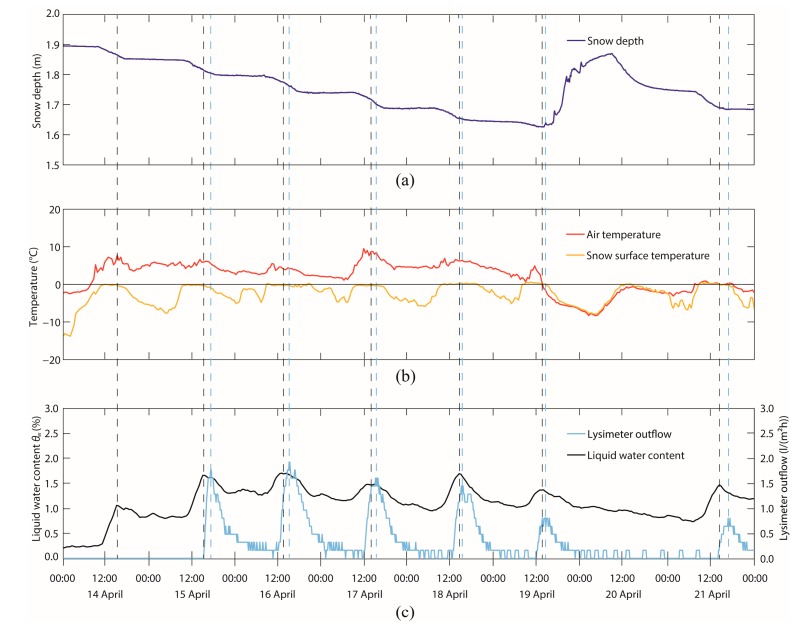
(**a**) Snow depth; (**b**) air and snow surface temperature and (**c**) lysimeter meltwater outflow, as well as the bulk volumetric LWC derived from normalized GPS C/N_0_ measurements at the Weissfluhjoch test site during the time period 14–21 April 2013. Daily peaks of the lysimeter outflow and the LWC are marked with the dashed lines in corresponding colors.

The daily evolution of GPS-derived LWC clearly showed the daily melt-freeze cycles typically observed during the snowmelt period. Figure 9 exemplarily shows the temporal evolution of measured snow depth, air and snow surface temperature and lysimeter meltwater outflow, as well as the LWC derived from the normalized GPS C/N_0_ measurements for eight days (14–21 April). During this period, the snow depth decreased in the first six days from 1.89 to 1.65 m. On the seventh day, it increased to 1.85 m during a snowfall and decreased again to 1.68 m on the eighth day. Snow depth decreased especially during mid-day when the snow surface temperature reached 0 °C and was stable at nighttime, in accordance with temperatures below 0 °C. During the first six days, the air temperature was almost always above 0 °C, and the snow surface temperature reached 0 °C during the daytime. During these days with melt activity, the bulk volumetric LWC showed a daily course, reaching a maximum in the afternoon. Furthermore, a time-lagged daily discharge maximum was observed. While reaching the maximum LWC, also the maximum percolation rate was reached within the snowpack. After the meltwater passed through the entire snowpack, it arrived at the bottom of the snowpack at the lysimeter with a delay of approximately 0.5 to 1.5 h after the daily LWC maximum was reached. During the end of the night and the early morning, the minima were reached due to refreezing processes. On 20 April, a dry snowfall was recorded, and the temperatures dropped to approximately 0 °C. Subsequently, no discharge was registered, and the LWC decreased. On the last day, a clear daily melt-freeze cycle can be observed again.

## 7. Discussion

### 7.1. Advantages and Limits

This new experimental low-cost GPS measurement system is capable of detecting bulk volumetric LWC continuously and non-destructively over an entire melting period. This means changes in the LWC, such as daily melt-freeze cycles, can be traced with half-hourly resolution. This highly temporally resolved and non-destructive information on the evolution of the LWC was so far only reached with radar systems [17,18]. Other *in situ* measurements, like dielectric probes or manual measurements, are all labor-intense, time consuming and invasive, which means that snow profiles have to be dug to derive snow pit information. Even though the LWC derived by GPS sensors can only deliver bulk values without information on the snow stratigraphy, the GPS data provide valuable information for hydrological applications, e.g., to detect the melt-onset with high temporal resolution. These GPS measurement systems have the clear advantage that they can be installed at large numbers, due to their cost efficiency, with low-cost devices and freely available GPS raw data. Sensor networks with large numbers of sensor members could be installed, monitoring on a large scale, e.g., melting processes of an entire hydrological catchment, or on a small scale, e.g., an avalanche prone slope, which is heterogeneously covered by snow. Nevertheless, for the LWC calculations, additional snow depth measurements are necessary. This could probably be overcome in the future by determining the snow depth by applying additional GPS measurements and algorithms (e.g., [25,27]).

### 7.2. Uncertainty Estimates for the Calculation of the Bulk Volumetric Liquid Water Content

For the GPS-derived LWC based on intensity losses, three common approaches exist to determine the real part of the complex permittivity for wet snow in the frequency range of the GPS signals. However, Figure 7 does not give an answer to which of the applied equations after Sihvola and Tiuri [15] (Equation (13)), Denoth [13] (Equation (14)) and Roth *et al.* [52] (Equation (16)) is the most realistic, though this is not subject of this study. Even if the curves of the bulk volumetric LWC lay close together, the choice of the mixing formula for wet snow may carry uncertainties in the derivation, especially if the wetness is high. For comparison with other data, the mean of the three quite common approaches was taken.

Besides differences in the results by applying the different empirical mixing formulas, further uncertainties remain. In addition to the normalized GPS C/N_0_ data, snow depth and the dry snow density are input parameters that may also be erroneous, e.g., due to spatial variations in snow depth, which may have implications on different Fresnel zones around the GPS antenna. Though the laser sensor is installed quite close to the GPS measurement setup, snow depth at the location of the GPS receivers can certainly deviate by a few centimeters. An overestimation in snow depth leads to an underestimation of the LWC and *vice versa*; the higher this error is, the lower is the snow depth. If, for example, the LWC is 4%, an overestimation of 10 cm in snow depth leads to *θ_w_* = 3.8% (*i.e.*, an underestimation by 5.6%) at a measured snow depth of 1.54 m at the laser sensor; if the measured snow depth is only 0.64 m, the same error in snow depth results in an underestimation of *θ_w_* by 13%. A possible effect of snow depth variability could, e.g., be reduced if the snow depth sensor would have been directly mounted above the GPS instruments, which was technically not feasible. However, errors in snow depth larger than about ±10 cm are rather unlikely at the test field, as further snow depth sensors and manual measurements showed.

The dry snow density was 370 kg/m^3^ before the snow became wet and was held constant over the entire melting period, as suggested by Mitterer *et al.* [8]. Deviations in dry snow density, for example due to settling during the melting period, have little effect on the calculation of the LWC. For example, for an LWC of 4.0%, a deviation in snow density of ±20% leads to an increase or decrease of the LWC to only 4.1 or 3.9%, respectively. To avoid uncertainties caused by the density assumption, it would only be possible to continuously derive the wet snow density, e.g., using independent measurements, which, however, could cause further measurement uncertainties.

Moreover, additional uncertainties may occur due to multiple reflection paths at and between the air/snow and snow/ground interfaces or at the flat hut or other poles at the test site and, in particular, affect the GPS antenna above the snow cover (GPS1). However, these effects are minor, compared to the considered reflection, refraction and attenuation processes at and within the snowpack, and these are neglected within this simple low-cost approach.

### 7.3. Comparison with Other Measurements

Few quantitative reference measurements were available. The bulk volumetric LWC derived by the non-destructive normalized GPS C/N_0_ measurements could only be compared to the few destructive measurements conducted with either the Denoth meter [13] or the Snow Fork [15] or to the wetness estimates for each stratigraphic layer according to Fierz *et al.* [10]. First, the destructive methods cannot be carried out at exactly the same location, including the problem that the snow wetness can spatially be variable. Second, they are all destructive methods, and due to the fact that the snow pit wall is exposed to atmospheric influences, the snow wetness can change rapidly and, hence, influence the measurements. Third, these reference measurements are quite labor-intense and, due to their destructiveness, were only performed on a few dates, so they are only a snapshot without showing the temporal evolution of the LWC. Forth, the manual measurements and observations within the snow pit are often subjective and can only be interpolated with limited accuracy for the entire snowpack. Fifth, the two dielectric methods show large discrepancies, which was already observed by Techel and Pielmeier [12], Mitterer *et al.* [8] and Schmid *et al.* [18].

Overall, we showed that the bulk volumetric LWC derived from GPS data was highly sensitive to the daily meteorological and snow-hydrological evolution and can plausibly be explained by them. Due to their frequent repetition and sensitivity, the measured C/N_0_ values from GPS signals can be used to resolve the evolution of the entire period and daily melt-freeze cycles and their impact on snow LWC. The daily evolution of the LWC derived from the GPS measurements was in good qualitative agreement with the daily evolution of the meteorological and snow-hydrological data, but we were at this point of our research unable to provide a proper quantitative validation, as appropriate *in situ* or remote sensing methods were not readily available.

## 8. Conclusion and Outlook

We presented a new approach to continuously determine snow liquid water content (LWC) with simple low-cost GPS receivers based on the attenuation in wet snow of the GPS signal strength broadcasted via L1-band microwaves. With this new experimental measurement approach, it was possible to directly derive from measured normalized GPS C/N_0_ data the bulk volumetric LWC of a seasonal snowpack continuously and non-destructively. Intensity losses due to reflection, refraction and attenuation processes within the atmosphere and the snowpack, as well as at the snow-atmosphere interface were considered.

The LWC was calculated with three common and plausible mixing formulas based on the real permittivity of wet snow at the high elevation Weissfluhjoch test site above Davos, Switzerland, over an entire melt period in spring, 2013. The GPS-derived temporal evolution of LWC was compared with single destructive reference measurements and continuous meteorological and snow-hydrological data. The LWC showed qualitatively a high temporal coincidence with the evolution of air and snow surface temperatures, snow depth and meltwater outflow during the entire observation period. Furthermore, the melt onset and daily melt-freeze cycles were clearly detected.

In summary, the comparison of the evolution of the meteorological and snow-hydrological data with the bulk volumetric LWC derived with GPS data showed that a wealth of information on the dynamics of snow LWC is contained in the measurements. The normalized GPS C/N_0_ values reacted very sensitively to an increase or decrease of the LWC within the snowpack, e.g., during melting and refreezing conditions, and provide an indication for meltwater outflow. However, it has to be clearly stated that with the available *in situ* reference measurements, it was not possible to quantitatively validate the measured snow LWC. Therefore, in a next step, the bulk volumetric LWC derived by normalized GPS C/N_0_ data should be compared with other continuous and non-destructive measurement methods for the same time period. A promising method for this task may be an upward-looking ground-penetrating radar system.

The main advantages of this approach are that the measurement devices are low cost and low power consuming and that data analysis is not time consuming and labor intensive. The GPS signals are freely, globally and continuously available, and through the development of dedicated GPS receivers for smart internet appliances, like smartphones, the market offers a wide range of highly-sensitive, cheap, small, robust and low-power devices. This means that the measurement principle can be applied on a global basis (provided power and telemetry is available) to acquire information on LWC at a high temporal resolution without destroying the snow cover. It is possible to join large numbers of these receivers to sensor networks, which, once installed within a hydrological catchment, could be used, e.g., to deliver data for predicting snow melt and runoff in their spatial distribution and at different elevation levels. This could help to validate and temporally complement remote sensing applications supporting continuous runoff predictions for flood or hydropower services. Moreover, due to the small size of the instruments and the non-destructive measurement setup, it is possible to install the GPS antennas also in steep slopes. This has the potential to, e.g., support avalanche forecasting through continuous information on snow wetness directly from avalanche-prone slopes, where, to date, this information is hardly available. Analyzing signal strength losses of normalized GPS C/N_0_ data caused by wet snow has therefore a high potential for continuously monitoring the LWC, e.g., in basin-wide sensor networks for hydrological applications or for avalanche predictions.

## Figures and Tables

**Table 1.** Real ${ɛ}_{s}^{\prime }$ and imaginary part ${ɛ}_{s}^{\prime \prime }$ of the complex permittivity of snow, corresponding reflectivity *r*^2^ and the attenuation coefficients *α* for varying values of LWC; LWC is the mean of the three approaches for the real part (Equations (13), (14) and (16)).

**Volumetric Liquid Water Content** ***θ***	**Permittivity, Real Part** ***ε*_s_*′***	**Permittivity, Imaginary Part** ***ε*_s_*′′***	**Reflectivity** ***r****^2^*	**Attenuation Coefficient** ***α***
0%	1.74	0.00	0.03	0.01
2%	2.10	0.04	0.05	0.83
4%	2.50	0.08	0.07	1.79
6%	2.96	0.14	0.09	2.75
8%	3.46	0.21	0.11	3.79

**Table 2.** Difference between the GPS-derived LWC and reference measurements (Denoth meter, Snow Fork and snow profile estimates). The absolute mean difference and the adjusted correlation coefficient are given.

	**Denoth Meter**	**Snow Fork**	**Snow Profile**
number of measurements	9	6	11
RMSD (pp)	1.0	1.2	1.2
adjusted R^2^	0.90	0.91	0.55
